# Differences in Lipid Metabolism between the Perirenal Adipose Tissue of Chinese Simmental Cattle and Angus Cattle (*Bos taurus*) Based on Metabolomics Analysis

**DOI:** 10.3390/ani14172536

**Published:** 2024-08-31

**Authors:** Siyuan Wang, Yue Pang, Lixiang Wang, Qi Wang, Zhongling Chen, Chengjiao Li, Fengjiao Li, Guoxi Zhang, Xiaoying Wang, Shuxin Gao, Xingjian Xu

**Affiliations:** 1 Hinggan League Agriculture and Animal Husbandry Science Institute, Ulanhot 137400, China; wangsiyuanme@126.com (S.W.); wangqi1995nys@163.com (Q.W.); chenzhongling3@163.com (Z.C.); xamxmyjsk@163.com (C.L.); momofengjiao@163.com (F.L.); nysxmsy@163.com (G.Z.); 2 College of Animal Science and Technology, Inner Mongolia Minzu University, Tongliao 028000, China; pangyue515@163.com (Y.P.); wanglixiang2022@163.com (L.W.); 3 Tongliao Animal Husbandry Development Center, Tongliao 028000, China; 13948146752@163.com

**Keywords:** Angus cattle, Simmental cattle, perirenal fat, lipid metabolism, metabolome

## Abstract

**Simple Summary:**

This study aimed to compare and analyze the metabolic differences in perirenal adipose tissue between Angus cattle and Chinese Simmental cattle. The results showed that the perirenal fat of Angus cattle had a larger cell area and stronger lipid deposition ability than that of Chinese Simmental cattle. Subsequently, 12 metabolites that may cause differences were screened out by liquid chromatography–tandem mass spectrometry technology. This study provides some basic data for further understanding the metabolic pattern of perirenal fat.

**Abstract:**

The aim of this experiment was to investigate the differences in metabolites in perirenal fat (PF) between Chinese Simmental cattle and Angus cattle. Six healthy 18-month-old male Angus cattle and Chinese Simmental cattle were selected, and the perirenal adipose tissue was collected after slaughtering. HE staining, a triglyceride assay kit, and liquid chromatography–tandem mass spectrometry (LC-MS/MS) technology were used to compare and analyze the differences in the cell morphology, lipid accumulation, and metabolites of the two types of PF. The results showed that the PF of Angus cattle had a larger cell area and stronger lipid deposition ability than that of Simmental cattle. A total of 567 metabolites were detected by LC-MS/MS technology, of which 119 were significantly upregulated in Angus cattle PF and 129 were significantly upregulated in Simmental cattle PF. Differential metabolites were enriched in pathways such as fatty acid biosynthesis, polyunsaturated fatty acid biosynthesis, regulation of adipocyte lipolysis, and oxidative phosphorylation. Finally, 12 metabolites that may cause phenotypic differences between the two types of perirenal adipose tissue were screened out from these pathways. This study has preliminarily screened out biomarkers that may affect lipid metabolism in PF, providing basic data for the further exploration of the metabolic characteristics of PF.

## 1. Introduction

Angus cattle (*Bos taurus*) raised in Chinese farms are purebred cattle that have been imported from Scotland in recent years. Due to their excellent meat quality, they are mainly used in the production of high-end beef [1]. Chinese Simmental is a hybrid between European Simmental and Chinese local yellow cattle. This species is a large dairy–meat hybrid with strong adaptability, cold tolerance, rough feeding resistance, and other characteristics [1]. Due to the early maturity, easy fattening ability, high feed conversion rate, and high intramuscular fat (IMF) content of Angus cattle, their meat quality is superior to that of Chinese Simmental cattle in terms of juiciness, tenderness, and flavor.

According to the distribution and deposition location, body fat can be divided into subcutaneous fat and visceral fat [2]. Subcutaneous adipose tissue is the largest fat storage reservoir in the body, but its storage capacity is limited. When its storage capacity is exceeded, fat is stored in ectopic fat reservoirs, such as the heart, liver, perirenal, and skeletal muscles, which are important contributors to obesity-associated diseases [3]. IMF content, as a direct indicator of beef quality, has always been a popular research topic. Li et al. [4] reported four key genes and SNP loci that affect IMF content in Japanese black cattle and Qinchuan cattle. Zheng et al. [1] used RNA-seq technology to screen for six candidate genes that may cause differences in IMF content between Simmental cattle and Angus cattle. Meanwhile, Yamada et al. [5] also detected a large number of differential metabolites among cattle breeds with different IMF contents. However, visceral fat is usually regarded as “waste” in beef production [6], which has led to most researchers overlooking its impact on the fat deposition and immune function of beef cattle. Recently, it has been reported that there is a positive correlation between perirenal fat (PF) and overall carcass fat in lambs [7]. PF is a special type of visceral fat that is stored around the kidney. The fibrous membrane and renal fascia separate PF from its adjacent adipose tissue (pararenal fat and renal sinus fat), which is active in the metabolic regulation and secretion of adipokines due to its unique anatomical structure and physiological characteristics [8]. To date, studies have shown that perirenal adipose tissue plays an important role in energy metabolism, adipokine biotransformation, and cytokine secretion [9]. However, the excessive accumulation of perirenal fat will increase the adipocyte size and activate the renin–angiotensin–aldosterone system, which will lead to adipose tissue dysfunction, inflammation, adipocyte apoptosis and imbalance of body metabolic homeostasis, eventually causing various metabolic syndromes, such as insulin resistance, type 2 diabetes, cardiovascular diseases, etc. [10,11]. In addition, a significant positive correlation between the size of perirenal adipocytes and body condition score was found in cows at the end of pregnancy (r = 0.90, *p* < 0.05) [12]. Moreover, the expression levels of interleukin-6 and tumor necrosis factor are higher in larger adipocytes (0.05 < *p* < 0.10) [12]. Through the above studies, we speculate that changes in the triglyceride content and cell size of bovine perirenal fat may cause changes in energy metabolism substances and immune-related active substances in bovine bodies.

Metabolomics uses high-throughput technology to detect endogenous metabolites in biological samples. Compared with traditional detection methods, it has characteristics such as high resolution and superior sensitivity [13]. In recent years, metabolomics technology has been widely used in screening biomarkers related to fat metabolism. Yamada et al. [5] used metabolomics technology to study the differences in metabolites in IMF between Japanese Wagyu (high IMF) and Holstein cattle (low IMF) and found that metabolites related to lipid synthesis, tricarboxylic acid cycle, and fatty acid metabolism were significantly upregulated in Japanese Wagyu cattle, while metabolites related to amino acid metabolism were significantly upregulated in Holstein cattle. Currently, little is known about the metabolic characteristics of visceral fat. Therefore, this study aims to use liquid chromatography–tandem mass spectrometry (LC-MS/MS) to compare and analyze the metabolites of two kinds of perirenal fat (high and low fat content) in order to screen out key biomarkers and provide some basic data for the further study of the metabolic pattern of perirenal fat.

## 2. Materials and Methods

### 2.1. Selection of Experimental Animals and Sample Collection

The animal study protocol was approved by the Experimental Animal Welfare and Ethics Committee of the College of Animal Science and Technology of Inner Mongolia Minzu University (protocol code: No. 2019089). All animal experiments were performed in accordance with the national standard titled the Guideline for Ethical Review of Animal Welfare (GB/T 35892–2018) [14].

The experimental animals used in this study were provided by Dumei Animal Husbandry Co., Ltd. (Tongliao, China). To avoid interference from age and disease factors, the screening criteria for experimental animals were as follows: (1) healthy and disease-free; (2) age difference between all experimental animals of less than 7 days; and (3) no significant difference in body weight among the groups. Six 18-month-old male Angus cattle (515.48 ± 8.89 kg) and six Chinese Simmental cattle (548.01 ± 7.63 kg) were selected. The experiment was conducted at Dumei Animal Husbandry Co., Ltd. in Tongliao, Inner Mongolia. All animals were semi-housed in the same free space, provided the same total mixed ration (TMR) shown in Table 1, and drank freely. After slaughter, 20 g of PF was collected from each cattle. All samples were immediately put into liquid nitrogen and preserved at −80 °C until later use.

### 2.2. Measurement of Adipocyte Area

The bovine PF tissue was fixed with 4% paraformaldehyde, embedded in paraffin, and sliced, followed by HE staining. Slice images were acquired using an ECLIPSE-CI fluorescence microscope (Nikon, Tokyo, Japan). The images were subsequently analyzed using Image Pro Plus 6.0 software (Media Cybernetics Corporation, Rockville, MD, USA). Four photos with different visual fields were selected, and four different visual fields were randomly selected for each photo. All the cells in each visual field were calculated. 

### 2.3. Determination of Triglyceride Content

The triglyceride content in PF was determined by using a high-fat sample triglyceride GPO-POD assay kit (E1025, Applygen, Beijing, China). A total of 20 mg of PF tissue was accurately weighed. Then, 1 mL of lysis solution was added, and the homogenate was allowed to stand for 10 min. Afterward, the sample was heated at 70 °C for 10 min in a water bath pot and centrifuged at 2000 rpm for 5 min. The supernatant was taken. Next, 10 μL of supernatant/standard triglycerides was taken, and 190 μL of working solution was added. The sample was left at room temperature for 15 min. Triglycerides were obtained by using a microtiter plate reader (Varioskan LUX, ThermoFisher Scientific, Waltham, MA, USA).

### 2.4. LC-MS/MS Detection

After pre-treatment, the samples were evaluated with liquid chromatography–tandem mass spectrometry (LC-MS/MS). The Hyperil Gold column (C18) chromatography column was used. The positive ion mode was as follows: mobile phase A was 0.1% formic acid, and mobile phase B was methanol. The negative ion mode was as follows: mobile phase A was 5 mmol/L ammonium acetate (pH 9.0), and mobile phase B was methanol. The column temperature was 40 °C, and the flow rate was 0.2 mL/min. The mass spectrometry conditions were as follows: a capillary temperature of 320 °C, a sheath gas pressure of 35 psi, and an auxiliary gas flow rate of 3 L/min. The electric spray ionization source conditions were as follows: a spray voltage of 3.2 kV and a scanning range of 100~1500 m/z.

### 2.5. Metabolomics Analysis

Firstly, the original files obtained from mass spectrometry detection were subjected to spectral processing and database comparison to obtain qualitative and quantitative results for metabolites. Then, the data were subjected to quality control. Subsequently, principal component analysis (PCA) and partial least squares discriminant analysis (PLS-DA) were performed on metabolites using Meta X [15]. Using the PLS-DA model, differential metabolites were identified by variable projection importance (VIP) and multiple of difference (FC) values, which were combined with the *p*-values from the *t*-tests. The screening criteria were VIP > 1.0, FC > 1.5, or FC < 0.667, with *p*-value < 0.05. The KEGG database was used to study the functions and metabolic pathways of differential metabolites, and *p* < 0.05 was used as the criterion for significant enrichment.

### 2.6. Statistical Analysis

Statistical analysis was performed using SPSS 27.0 software (International Business Machines Corporation, Armonk, NY, USA). Student′s *t*-test was used to evaluate the differences in adipocyte area, quantity, and triglyceride content between the PF samples of Simmental cattle and Angus cattle. Experimental data were presented as means ± SEM. The differences were considered statistically significant at *p* < 0.05.

## 3. Results

### 3.1. Observation of Organizational Morphology and Determination of Lipid Content

**Figure 1:**
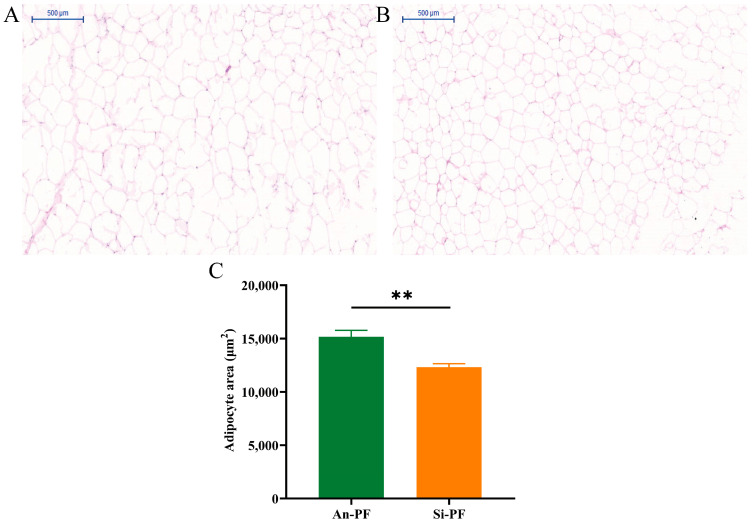
Histomorphological observations of PF in Angus cattle (**A**) and Simmental cattle (**B**); (**C**) average area of a single cell in adipose tissue. The values are represented as means ± SEM. ** *p* < 0.01.

**Figure 2:**
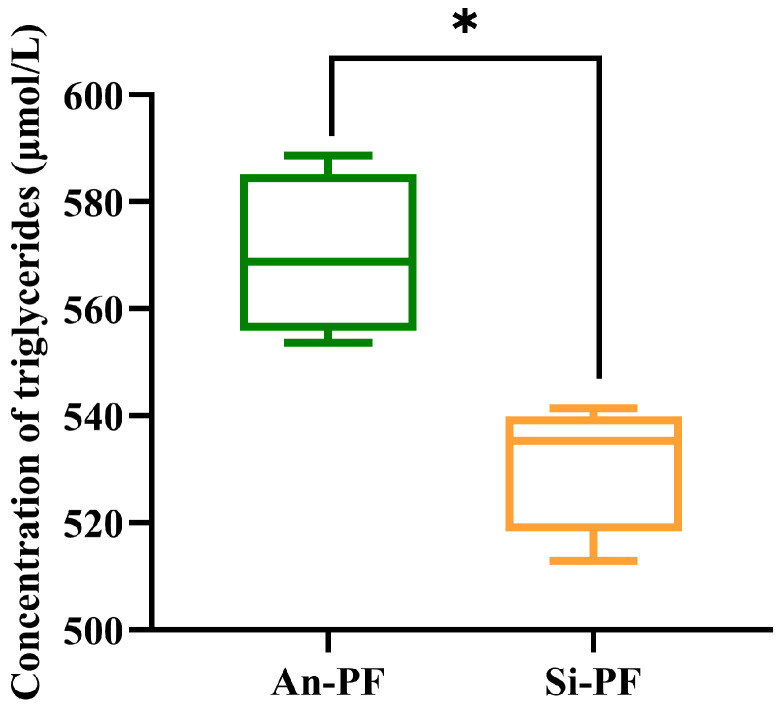
Levels of triglycerides in PF of Angus and Simmental cattle. The values are represented as means ± SEM. * *p* < 0.05.

The tissue section results of adipose tissue are shown in Figure 1. The results showed that the cell area of the Angus cattle PF tissue was significantly higher than that of the Simmental cattle PF tissue (*p* < 0.01), as shown in Figure 1C. In addition, the results of the triglyceride assay kit showed that the concentration of triglycerides in the PF of Angus cattle was significantly higher than that in the Simmental cattle, as shown in Figure 2. The above results preliminarily revealed significant differences in the tissue morphology and fat deposition ability of the two types of PF.

### 3.2. Metabolite Identification and Principal Component Analysis

**Figure 3:**
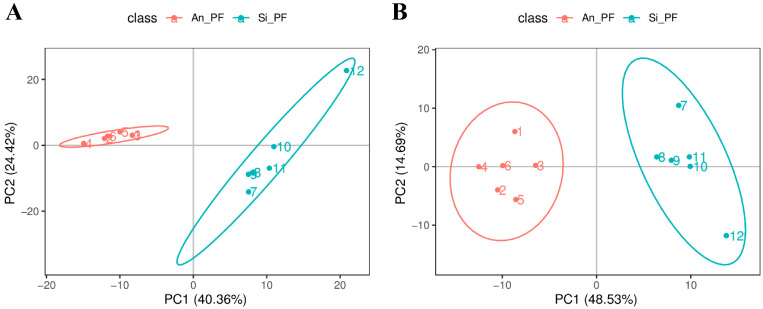
Principal component analysis: (**A**) positive ion mode; (**B**) negative ion mode. The red circle area represents Angus perirenal fat, and the blue area represents Chinese Simmental perirenal fat. Numbers represent sample numbers for each group.

A total of 567 metabolites were detected in the 12 samples analyzed in this study, with 357 and 210 metabolites detected in the positive and negative ion modes, respectively. Using the PCA method, the overall distribution trends between the two groups of samples were observed. The results showed that both groups of samples could be effectively clustered together (Figure 3). This PCA effectively showed the differences in the metabolites between the two groups, which met the requirements of the subsequent analysis.

### 3.3. Partial Least Squares Discrimination Analysis

**Figure 4:**
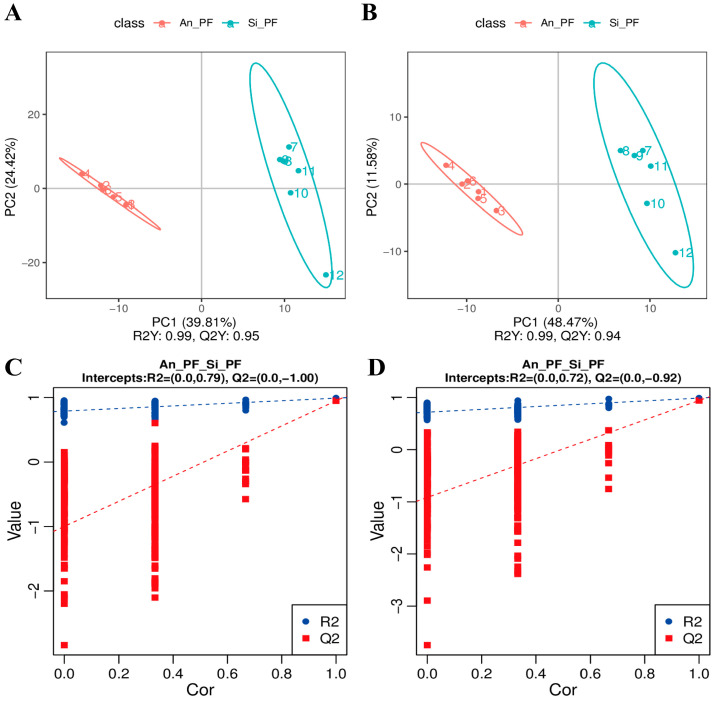
OPLS-DA score plot: (**A**) positive ion mode; (**B**) negative ion mode. Permutation tests: (**C**) positive ion mode; (**D**) negative ion mode.

In the PLS-DA model (Figure 4A,B), the two groups of samples were clearly distinguished, indicating significant differences in metabolites in PF between Angus cattle and Simmental cattle. The permutation test results in Figure 4C,D show that as the correlation coefficient decreases (permutation retention decreases), Q2 and R2 gradually decrease. The Q2 regression curve intersects with the y-axis below 0 and R2 > Q2, indicating that the constructed model has passed the permutation test without overfitting and can be used for screening differential metabolites.

### 3.4. Screening of Differential Metabolites

**Figure 5:**
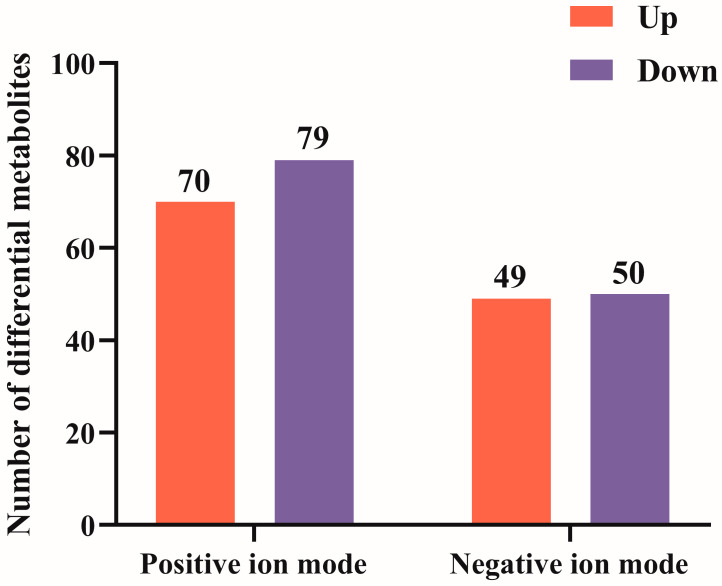
Number of differential metabolites.

**Figure 6:**
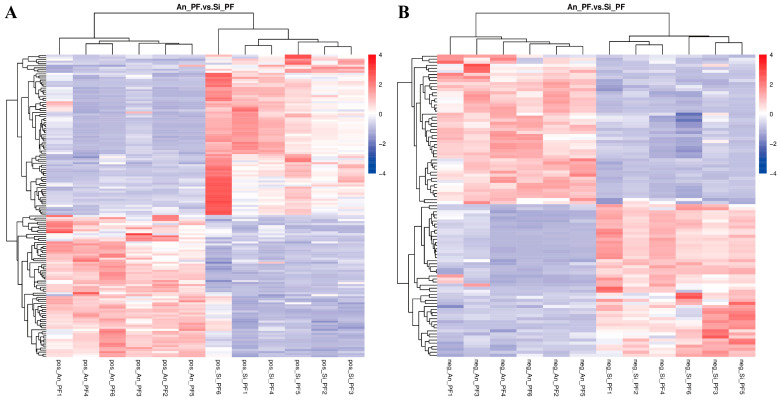
Cluster heatmaps of differential metabolites under the positive (**A**) and negative ion modes (**B**).

According to the VIP value of the first principal component of the PLS-DA model, 248 differential metabolites were obtained after screening according to the criteria of VIP > 1.0, FC > 1.5 or FC < 0.667, and *p*-value < 0.05. Compared with Simmental cattle, the relative concentrations of 119 metabolites in the PF of Angus cattle were significantly increased, while 129 metabolites were significantly decreased (Figure 5 and Tables S1 and S2). Subsequently, the differential metabolites in the positive and negative ion modes were clustered separately. The results showed that these differential metabolites could be used to clearly distinguish the PF samples from Angus cattle and Simmental cattle. The metabolites with similar metabolic patterns were clustered in the same cluster (Figure 6), indicating that the cluster differentiation of metabolites between the groups was obvious.

### 3.5. Enrichment Pathway Analysis of Differential Metabolites 

In order to study the biological pathways involved in differential metabolites and the correlations between metabolites, pathway enrichment analysis was performed on the 248 differential metabolites screened. The results showed that the differential metabolites were enriched in lipid metabolism-related pathways such as fatty acid biosynthesis, polyunsaturated fatty acid biosynthesis, regulation of adipocyte lipolysis, oxidative phosphorylation and fatty acid metabolism. Further screening of metabolites closely related to fat metabolism and immune function in the pathway revealed that eight metabolites—oleic acid (OA), palmitoleic acid (POA), palmitic acid (PA), arachidonic acid (ARA), prostaglandin H2, docosahexaenoic acid (DHA), docosapentaenoic acid (DPA), and eicosapentaenoic acid (EPA)—were significantly upregulated in Angus PF. The contents of the four metabolites—adrenaline, NAD^+^, NADH, and spermidine—were significantly upregulated in the PF of Simmental cattle, as shown in Table 2.

## 4. Discussion

Visceral fat is closely related to energy metabolism and immune function in the body. In this study, through the comparative analysis of tissue morphology and the metabolome of perirenal adipose tissue with a high fat content (Angus cattle) and a low fat content (Chinese Simmental cattle), we found some phenotypic differences between the two types of perirenal fat and screened out a large number of differential metabolites related to fat metabolism and immune function, which provided valuable information for understanding the metabolic characteristics of PF.

### 4.1. Effects of Different Metabolites on Perirenal Fat Metabolism

We found that metabolites related to fatty acid biosynthesis pathways (OA, POA, and PA) and polyunsaturated fatty acid biosynthesis pathways (ARA, prostaglandin H2, DHA, DPA, and EPA) were significantly upregulated in high-fat PF (Angus cattle). Fatty acids are an important component of triglycerides (TAGs) in adipose tissue and can actively participate in the regulation of TAG generation [16]. OA, as the main monounsaturated fatty acid (MUFA) in the body′s circulation, can be produced through diet and endogenous synthesis. It has been widely shown that OA plays an important role in the synthesis of intracellular TAGs [17,18]. Tsuchiya et al. [19] found that OA in adipocytes can activate the insulin receptor/PI3K/PDK1/AKt/Rac1 pathway by inhibiting the activity of protein tyrosine phosphatase non-receptor type 1 (*PTP1B*), thereby enhancing insulin receptor signaling and promoting the insulin-induced increase in intracellular TAG synthesis. Meanwhile, Regassa et al. [20] and Malodobar et al. [21] both found that OA can stimulate the expression levels of lipogenic genes, such as peroxisome proliferator-activated receptor gamma (*PPARγ*), CCAAT enhancer binding protein alpha (*C/EBPα*), and fatty acid binding protein 4 (*FABP4*) in preadipocytes, thereby promoting the adipogenesis and differentiation of preadipocytes. The increases in *PPARγ* and *CEAPα* expression may be related to the decrease in the promoter methylation level. In addition, OA can also promote the expression level of peroxisome proliferator-activated receptor gamma coactivator 1beta (*PGC-1β*) protein in skeletal muscle, enhance the synthesis of TAGs, and transform the type of fiber to 2X [22]. According to the above studies, OA can promote the synthesis of intracellular TAGs through multiple pathways. POA is one of the most abundant fatty acids in adipose tissue; it is produced by stearoyl-Co A desaturase 1 (*SCD1*) catalyzing PA [23]. Since POA is released from adipose tissue and has a regulatory effect on the metabolism of other organs, it is regarded as a “lipid factor” [24]. Gong et al. [25] observed a strong positive correlation between the concentration of POA in adipose tissue and the occurrence of obesity. Moreover, studies have shown that abdominal fat accumulation is positively correlated with the concentration of POA in plasma, which is consistent with the enhancement of *SCD1* activity [26]. In addition, POA, as a “lipid factor”, can also mediate the connection between adipose tissue and the liver, inhibiting the activity of *SCD1* and the deposition of TAGs in the liver [24]. Cruz et al. [27] found that POA promoted the synthesis of TAGs in subcutaneous and visceral adipose tissues but inhibited the increase in TAGs in the liver induced by a high-fat diet (HFD) by treating HFD mice with POA. Therefore, the increase in POA concentration in adipose tissue may promote the accumulation of TAGs. As the precursor of various fatty acids, PA is the most common saturated fatty acid in the body, accounting for approximately 20% to 30% of the total fatty acids in TAGs. Generally, the body can prevent the accumulation of PA by enhancing the synthesis of POA, stearic acid (SA), and OA so that its concentration in various tissues remains stable [28]. However, excessive PA can lead to the dysregulation of fat deposition and distribution [28]. Polyunsaturated fatty acids (PUFAs) are structural components of cell membrane phospholipids which can exert their biological functions by affecting membrane properties and acting as precursors for various lipid mediators; PUFAs are mainly divided into *n*-3 PUFA and *n*-6 PUFA [29]. ARA is an important *n*-6 PUFA and a direct precursor of eicosanoid bioactive mediators (prostaglandin, leukotriene, and eicosatrienoic acid), while DHA, DPA, and EPA are three well-known *n*-3 PUFAs [30]. Numerous studies have shown that *n*-6 PUFA and its formed lipid mediators have pro-inflammatory potential, while *n*-3 PUFA exhibits anti-inflammatory properties [31,32]. The ARA and its derivatives have strong biological activities, which can amplify the inflammatory response through the p38 MAPK signaling pathway [33]. At the same time, ARA can also stimulate lipogenesis by activating *PPARγ*, inhibit macrophage M2 polarization, and enhance the inflammatory response [34,35]. The anti-inflammatory effect of *n*-3 PUFA can be mediated by acting as a competitive substrate for *n*-6 PUFA metabolism or by producing specific pro-inflammatory and anti-inflammatory mediators [32]. In addition, *n*-3 PUFA can also inhibit the downstream cascade reaction of ARA, thereby attenuating inflammation [36]. Based on the above research, we speculate that the upregulation of OA, POA, PA, ARA, and prostaglandin H2 may promote the synthesis of TAGs and induce an inflammatory response in PF, while increases in the DHA, EPA, and DPA contents may occur to prevent the further exacerbation of the inflammatory response.

In low-fat PF (Simmental cattle), we observed that the contents of adrenaline, NAD^+^, NADH, and spermidine were significantly upregulated. Adrenaline is released from the adrenal medulla and mainly drives catabolism. Adrenaline can bind to the membrane receptors of the G protein-coupled receptor superfamily in target cells, where β3 adrenergic receptors (β-3AR) play an important role in adipose tissue [37]. After binding with β-3AR, adrenaline activates a signaling cascade reaction, leading to increases in intracellular cAMP levels and the activation of AMPK, thereby promoting the breakdown of TAGs [38]. Moreover, Hattori et al. [39] observed enhanced AMPK activation in visceral fat in a rat model with high epinephrine levels, further confirming this viewpoint. Furthermore, enhanced phosphorylation of acetyl-CoA carboxylase (ACC) has been observed; both β-3AR inhibitors and AMPK inhibitors can block ACC phosphorylation [39]. These results indicate that high levels of adrenaline can induce ACC phosphorylation through the β-3AR and AMPK pathways to reduce visceral fat accumulation. NAD^+^/NADH is an important redox pair in mitochondria that is involved in mitochondrial respiratory catabolism. NAD^+^ is reduced to NADH after receiving the hydride ion (H^+^) from the reactants, and it oxidizes the reactants to generate energy. Studies have shown that the levels of NDA^+^ in white adipose tissue decrease during a period of obesity [40]. Similarly, long-term HFD also reduces NAD content in subcutaneous fat, visceral fat, and brown fat [41]. In addition, supplementation of nicotinamide riboside (NAD^+^ precursor) to mice fed with HFD can increase NAD^+^ levels and activate Sirtuin 1 and Sirtuin 3, thereby enhancing oxidative metabolism and reducing TAG synthesis induced by HFD [42]. Spermidine is a natural antioxidant stress agent and autophagy inducer which has physiological functions such as stabilizing nucleic acid, improving metabolism, promoting cell proliferation, and improving immunity [43]. Exogenous spermidine supplements can increase the lifespan of humans and mice and improve age-related diseases [44,45]. Based on the above research, we speculate that the upregulation of adrenaline, NAD^+^, NADH, and spermidine may inhibit the synthesis of TAGs in PF.

### 4.2. Limitations of This Study

In this study, LC-MS/MS technology was used to analyze the metabolites of perirenal fat in Angus cattle and Chinese Simmental cattle. We need to emphasize that fat metabolism is a complex biological process that is comprehensively regulated by multiple factors and pathways. Although the same feeding management measures and diet composition were adopted for both Angus and Chinese Simmental cattle in this study, due to the inability to completely control the feed intake of each experimental animal, the differences observed during the experimental process may not fully reflect the inherent differences between the breeds and may also be influenced by these uncontrollable factors.

## 5. Conclusions

In this study, we found that the PF of Angus cattle has a larger cell area and stronger lipid deposition ability than that of Simmental cattle. In addition, 248 different metabolites were identified between the two types of PF by LC-MS/MS technology, among which 12 differential metabolites, such as OA, POA, PA, ARA, prostaglandin H2, DHA, DPA, EPA, adrenaline, NAD^+^, NADH, and spermidine, may be biomarkers that cause differences in cell morphology and fat metabolism between the two kinds of PF.

## Figures and Tables

**Table 1:** TMR composition and nutrient composition (% of dry matter).

Ingredients	Content/%	Nutrient Composition	Content/%
Straw	25.00	NEmf (MJ/kg)	7.01
Corn silage	25.00	Crude protein	13.11
Corn	25.00	Neutral detergent fiber	34.91
Bran	9.00	Acid detergent fiber	19.88
Soybean meal	7.00	Hemicellulose	16.59
Cottonseed meal	6.00	Cellulose	16.42
Urea	0.60	Crude ash	8.44
CaCO_3_	0.75	Acid detergent lignin	2.39
NaCl	0.55		
NaHCO_3_	0.35		
CaHPO_4_	0.25		
Premix ^1^	0.50		
Total	100.00		

^1^ The premix provided the following per kg of the diet: Fe 50 mg, Cu 10 mg, Mn 20 mg, Zn 30 mg, I 0.5 mg, Se 0.1 mg, VA 1500 IU, VD 550 IU, and VE 10 IU.

**Table 2:** Differential metabolites and their related parameters.

Differential Metabolites	FC	VIP	*p*-Value	Up–Down Regulation ^1^
Oleic acid	4.91	1.58	*p* < 0.01	Up
Palmitoleic acid	3.25	1.49	*p* < 0.01	Up
Palmitic acid	2.46	1.05	*p* = 0.02	Up
Arachidonic acid	2.98	1.15	*p* = 0.01	Up
Prostaglandin H2	2.46	1.39	*p* < 0.01	Up
Docosahexaenoic acid	4.13	1.19	*p* = 0.01	Up
Docosapentaenoic acid	6.99	1.43	*p* < 0.01	Up
Eicosapentaenoic acid	1.70	1.28	*p* < 0.01	Up
Adrenaline	0.13	1.31	*p* < 0.01	Down
NAD^+^	0.28	1.35	*p* < 0.01	Down
NADH	0.32	1.06	*p* = 0.01	Down
Spermidine	0.19	1.37	*p* < 0.01	Down

^1^ “Up–down regulation” refers to the up- or downregulation of metabolites in Angus cattle PF compared with that in Simmental cattle PF samples.

## Data Availability

The data that support the findings of this study are available from the corresponding authors upon reasonable request.

## Supplementary Materials

The following supporting information can be downloaded at https://www.mdpi.com/article/10.3390/ani14172536/s1. Table S1: Differential metabolites of perirenal fat between Angus cattle and Simmental cattle (positive ion mode). Table S2: Differential metabolites of perirenal fat between Angus cattle and Simmental cattle (negative ion mode).

## References

[1] Zheng Y., Chen J., Wang X., Han L., Yang Y., Wang Q., Yu Q. (2022). Metagenomic and transcriptomic analyses reveal the differences and associations between the gut microbiome and muscular genes in Angus and Chinese Simmental cattle. Front. Microbiol..

[2] Ibrahim M.M. (2010). Subcutaneous and visceral adipose tissue: Structural and functional differences. Obes. Rev..

[3] Smith U., Kahn B.B. (2016). Adipose tissue regulates insulin sensitivity: Role of adipogenesis, denovo lipogenesis and novel lipids. J. Intern. Med..

[4] Li Y., Cheng G., Yamada T., Liu J., Zan L., Tong B. (2020). Effect of expressions and SNPs of candidate genes on intramuscular fat content in Qinchuan cattle. Animals.

[5] Yamada T., Kamiya M., Higuchi M. (2022). Metabolomic analysis of plasma and intramuscular adipose tissue between Wagyu and Holstein cattle. J. Vet. Med. Sci..

[6] Yang X., Zhu R., Song Z., Shi D., Huang J. (2023). Diversity in cell morphology, composition, and function among adipose depots in river buffaloes. Int. J. Mol. Sci..

[7] Alonso-García M., Suárez-Vega A., Fonseca P.A.S., Marina H., Pelayo R., Mateo J., Arranz J.J., Gutiérrez-Gil B. (2023). Transcriptome analysis of perirenal fat from Spanish Assaf suckling lamb carcasses showing different levels of kidney knob and channel fat. Front. Vet. Sci..

[8] Jiang M., Li M., Liu C., Jing L., Huang Q., Wu T., Kong X., Liu J. (2022). Perirenal fat volume is positively associated with serum uric acid levels in Chinese adults. Front. Endocrinol.

[9] Xia S., Shao J., Elzo M.A., Tang T., Li Y., Lai T., Gan M., Ma Y., Jia X., Lai S. (2021). Untargeted metabolomics analysis revealed lipometabolic disorders in perirenal adipose tissue of rabbits subject to a high-fat diet. Animals.

[10] Liu Y., Wang L., Luo M., Chen N., Deng X., He J., Zhang L., Luo P., Wu J. (2019). Inhibition of PAI-1 attenuates perirenal fat inflammation and the associated nephropathy in high-fat diet-induced obese mice. Am. J. Physiol. Endocrinol. Metab..

[11] Foster M.T., Pagliassotti M.J. (2012). Metabolic alterations following visceral fat removal and expansion: Beyond anatomic location. Adipocyte.

[12] Depreester E., De Koster J., Van Poucke M., Hostens M., Van den Broeck W., Peelman L., Contreras G.A., Opsomer G. (2018). Influence of adipocyte size and adipose depot on the number of adipose tissue macrophages and the expression of adipokines in dairy cows at the end of pregnancy. J. Dairy Sci..

[13] Sun H.Z., Wang D.M., Wang B., Wang J.K., Liu H.Y., Guan le L., Liu J.X. (2015). Metabolomics of four biofluids from dairy cows: Potential biomarkers for milk production and quality. J. Proteome. Res..

[14] (2018). Guidelines for Ethical Review of Laboratory Animal Welfare.

[15] Wen B., Mei Z., Zeng C., Liu S. (2017). metaX: A flexible and comprehensive software for processing metabolomics data. BMC Bioinform..

[16] Yanting C., Yang Q.Y., Ma G.L., Du M., Harrison J.H., Block E. (2018). Dose- and type-dependent effects of long-chain fatty acids on adipogenesis and lipogenesis of bovine adipocytes. J. Dairy Sci..

[17] Listenberger L.L., Han X., Lewis S.E., Cases S., Farese R.V., Ory D.S., Schaffer J.E. (2003). Triglyceride accumulation protects against fatty acid-induced lipotoxicity. Proc. Natl. Acad. Sci. USA.

[18] Piccinin E., Cariello M., De Santis S., Ducheix S., Sabbà C., Ntambi J.M., Moschetta A. (2019). Role of oleic acid in the gut-liver axis: From diet to the regulation of its synthesis via Stearoyl-CoA Desaturase 1 (SCD1). Nutrients.

[19] Tsuchiya A., Nagaya H., Kanno T., Nishizaki T. (2014). Oleic acid stimulates glucose uptake into adipocytes by enhancing insulin receptor signaling. J. Pharmacol. Sci..

[20] Regassa A., Kim W.K. (2013). Effects of oleic acid and chicken serum on the expression of adipogenic transcription factors and adipogenic differentiation in hen preadipocytes. Cell. Biol. Int..

[21] Malodobra-Mazur M., Cierzniak A., Dobosz T. (2019). Oleic acid influences the adipogenesis of 3T3-L1 cells via DNA Methylation and may predispose to obesity and obesity-related disorders. Lipids. Health Dis..

[22] Komiya Y., Iseki S., Ochiai M., Takahashi Y., Yokoyama I., Suzuki T., Tatsumi R., Sawano S., Mizunoya W., Arihara K. (2024). Dietary oleic acid intake increases the proportion of type 1 and 2X muscle fibers in mice. Sci. Rep..

[23] Frigolet M.E., Gutierrez-Aguilar R. (2017). The role of the novel lipokine palmitoleic acid in health and disease. Adv. Nutr..

[24] Cao H., Gerhold K., Mayers J.R., Wiest M.M., Watkins S.M., Hotamisligil G.S. (2008). Identification of a lipokine, a lipid hormone linking adipose tissue to systemic metabolism. Cell.

[25] Gong J., Campos H., McGarvey S., Wu Z., Goldberg R., Baylin A. (2011). Adipose tissue palmitoleic acid and obesity in humans: Does it behave as a lipokine?. Am. J. Clin. Nutr..

[26] Paillard F., Catheline D., Duff F.L., Bouriel M., Deugnier Y., Pouchard M., Daubert J.C., Legrand P. (2008). Plasma palmitoleic acid, a product of stearoyl-coA desaturase activity, is an independent marker of triglyceridemia and abdominal adiposity. Nutr. Metab. Cardiovasc. Dis..

[27] Cruz M.M., Simão J.J., de Sá R.D.C.C., Farias T.S.M., da Silva V.S., Abdala F., Antraco V.J., Armelin-Correa L., Alonso-Vale M.I.C. (2020). Palmitoleic acid decreases non-alcoholic hepatic steatosis and increases lipogenesis and fatty acid oxidation in adipose tissue from obese mice. Front. Endocrinol..

[28] Carta G., Murru E., Banni S., Manca C. (2017). Palmitic acid: Physiological role, metabolism and nutritional implications. Front. Physiol..

[29] Dyall S.C., Balas L., Bazan N.G., Brenna J.T., Chiang N., da Costa Souza F., Dalli J., Durand T., Galano J.M., Lein P.J. (2022). Polyunsaturated fatty acids and fatty acid-derived lipid mediators: Recent advances in the understanding of their biosynthesis, structures, and functions. Prog. Lipid. Res..

[30] Duan J., Song Y., Zhang X., Wang C. (2021). Effect of omega-3 polyunsaturated fatty acids-derived bioactive lipids on metabolic disorders. Front. Physiol..

[31] Wiktorowska-Owczarek A., Berezinska M., Nowak J.Z. (2015). PUFAs: Structures, metabolism and functions. Adv. Clin. Exp. Med..

[32] Harwood J.L. (2023). Polyunsaturated fatty acids: Conversion to lipid mediators, roles in inflammatory diseases and dietary sources. Int. J. Mol. Sci..

[33] Zhang Y., Liu Y., Sun J., Zhang W., Guo Z., Ma Q. (2023). Arachidonic acid metabolism in health and disease. MedComm (2020).

[34] Xu M., Wang X., Li Y., Geng X., Jia X., Zhang L., Yang H. (2021). Arachidonic acid metabolism controls macrophage alternative activation through regulating oxidative phosphorylation in PPARgamma dependent manner. Front. Immunol..

[35] Demmelmair H., Koletzko B. (2021). Perinatal polyunsaturated fatty acid status and obesity risk. Nutrients.

[36] Lands B. (2015). Omega-3 PUFAs lower the propensity for arachidonic acid cascade overreactions. Biomed. Res. Int..

[37] Zhang D., Wei Y., Huang Q., Chen Y., Zeng K., Yang W., Chen J., Chen J. (2022). Important Hormones regulating lipid metabolism. Molecules.

[38] Omar B., Zmuda-Trzebiatowska E., Manganiello V., Göransson O., Degerman E. (2009). Regulation of AMP-activated protein kinase by cAMP in adipocytes: Roles for phosphodiesterases, protein kinase B, protein kinase A, Epac and lipolysis. Cell. Signal..

[39] Hattori A., Mawatari K., Tsuzuki S., Yoshioka E., Toda S., Yoshida M., Yasui S., Furukawa H., Morishima M., Ono K. (2010). Beta-adrenergic-AMPK pathway phosphorylates acetyl-CoA carboxylase in a high-epinephrine rat model, SPORTS. Obesity.

[40] Rappou E., Jukarainen S., Rinnankoski-Tuikka R., Kaye S., Heinonen S., Hakkarainen A., Lundbom J., Lundbom N., Saunavaara V., Rissanen A. (2016). Weight loss is associated with increased NAD(+)/SIRT1 expression but reduced PARP activity in white adipose tissue. J. Clin. Endocrinol. Metab..

[41] Wei X., Jia R., Wang G., Hong S., Song L., Sun B., Chen K., Wang N., Wang Q., Luo X. (2020). Depot-specific regulation of NAD(+)/SIRTs metabolism identified in adipose tissue of mice in response to high-fat diet feeding or calorie restriction. J. Nutr. Biochem..

[42] Cantó C., Houtkooper R.H., Pirinen E., Youn D.Y., Oosterveer M.H., Cen Y., Fernandez-Marcos P.J., Yamamoto H., Andreux P.A., Cettour-Rose P. (2012). The NAD(+) precursor nicotinamide riboside enhances oxidative metabolism and protects against high-fat diet-induced obesity. Cell. Metab..

[43] Jiang D., Wang X., Zhou X., Wang Z., Li S., Sun Q., Jiang Y., Ji C., Ling W., An X. (2023). Spermidine alleviating oxidative stress and apoptosis by inducing autophagy of granulosa cells in Sichuan white geese. Poult. Sci..

[44] Kiechl S., Pechlaner R., Willeit P., Notdurfter M., Paulweber B., Willeit K., Werner P., Ruckenstuhl C., Iglseder B., Weger S. (2018). Higher spermidine intake is linked to lower mortality: A prospective population-based study. Am. J. Clin. Nutr..

[45] Ni Y.Q., Liu Y.S. (2021). New insights into the roles and mechanisms of spermidine in aging and age-related diseases. Aging. Dis..

