# Supplementary material for: Differences in Lipid Metabolism between the Perirenal Adipose Tissue of Chinese Simmental Cattle and Angus Cattle (Bos taurus) Based on Metabolomics Analysis
Source: Animals (Basel). 2024 Aug 31;14(17):2536. doi: 10.3390/ani14172536 (PMC11394394; doi:10.3390/ani14172536)
Supplement: Supplementary file 1 [file animals-14-02536-s001.zip › animals-3145290-supplementary.pdf]

Table S1. Differential metabolites of perirenal fat between Angus cattle and Simmental cattle(Positive ion mode).

Name	RT [min]	m/z	FC	log2FC	Pvalue	VIP	Up.Down1)
LPE 20:3	9.242	504.30765	7.700250378	2.944905357	0.00	1.593873436	up
1-(4-benzylpiperazino)-2-(pyridin-2-ylamino)propan-1-one	9.56	325.20099	6.349372193	2.66661395	0.00	1.591372948	up
Oleic acid	8.802	283.26257	4.910268241	2.295801839	0.00	1.57891573	up
Prostaglandin A3	9.242	315.19266	3.952311736	1.982696743	0.00	1.5759815	up
Progesterone	7.269	315.23129	9.304297906	3.21789729	0.00	1.563880583	up
2-Oxindole	5.564	134.05981	0.045394505	-4.461338523	0.00	1.557240461	down
N-Phenylacetylglutamine	5.314	265.11783	0.106131099	-3.236080628	0.00	1.552462267	down
Ethyl oleate	9.612	311.29382	3.239993849	1.695991074	0.00	1.573089697	up
Tetranor-12(S)-HETE	8.094	249.18221	4.689890028	2.229554094	0.00	1.53007853	up
Epitestosterone	6.726	289.21579	0.140817495	-2.828101514	0.00	1.531347443	down
(5-methyl-3-isoxazolyl)[4-(5-propyl-2-pyrimidinyl)piperazino]methanone	6.3	316.1748	0.206205992	-2.27784184	0.00	1.532390032	down
benzoylecgonine-d3	5.077	293.16031	0.151644961	-2.721230534	0.00	1.53125032	down
ACar 20:3	7.271	450.35742	4.058672434	2.021007908	0.00	1.52083208	up
Prolylleucine	1.353	229.15445	0.22714	-	0.00	1.52523	down

			7258	2.1383 00202		4401	
UDP	1.664	405.00861	0.15933 5297	- 2.6498 62193	0.00	1.56549 0116	down
2-[(3S)-1-(2-Methylbenzyl)-3-pyrrolidinyl]-1H-imidazo[4,5-b]pyridine	9.85	293.17813	2.39331 3198	1.2590 09206	0.00	1.51965 9635	up
L-Ergothioneine	1.347	230.09552	9.54517 1189	3.2547 71072	0.00	1.54321 7703	up
5'-Deoxy-5'-(Methylthio)Adenosine	5.057	298.09631	0.26547 4407	- 1.9133 5531	0.00	1.50787 6923	down
16-Hydroxyhexadecanoic acid	7.893	273.24188	3.73723 9281	1.9019 72937	0.00	1.53450 2327	up
9-Oxo-ODE	9.499	295.22592	3.29871 7391	1.7219 05184	0.00	1.54454 0336	up
(2E,4E)-N-[2-(4-hydroxyphenyl)ethyl]dodeca-2,4-dienamide	4.773	316.22256	0.29957 7939	- 1.7389 96709	0.00	1.49464 848	down
PC (18:3e/2:0)	9.265	546.3548	7.65708 2881	2.9367 94873	0.00	1.54339 4349	up
Palmitoleic acid	8.032	255.23134	3.24837 3955	1.6997 17726	0.00	1.49310 5789	up
1,4-dihydroxyheptadec-16-en-2-yl acetate	8.129	311.25739	2.76916 928	1.4694 53249	0.00	1.52220 1689	up
11β-Hydroxyandrosterone	8.933	324.25809	3.321111 862	1.7316 66317	0.00	1.50517 1116	up
N2,N2-Dimethylguanosine	4.879	312.1297	0.19491 6136	- 2.3590 74568	0.00	1.49778 9763	down
All-Trans-13,14-Dihydroretinol	10.476	289.25217	2.76669 3112	1.4681 62626	0.00	1.47952 313	up

DLK	10.268	375.2262	7.821365783	2.967420556	0.00	1.48446402	up
Artemisinin	6.039	300.18011	0.15348879	-2.703794805	0.00	1.46976452	down
(+/-)12(13)-DiHOME	8.133	297.24161	2.756073771	1.462614505	0.00	1.537400536	up
(R)-3-Hydroxy myristic acid	7.405	227.20006	3.468473619	1.794300911	0.00	1.513301039	up
Adenosine	1.343	268.1033	0.394845149	-1.34064113	0.00	1.4514159	down
PC (18:4e/2:0)	8.876	544.33954	3.120660825	1.641851564	0.00	1.48390081	up
2-hydroxy-6-[(8Z,11Z)-pentadeca-8,11,14-trien-1-yl]benzoic acid	10.156	325.21091	2.163422637	1.113315532	0.00	1.466360891	up
Acetylcarnitine	5.621	204.12288	0.145672379	-2.77920074	0.00	1.493233942	down
Ciprostene	9.628	347.26352	1.786549691	0.837176042	0.00	1.437423918	up
13-HPODE	8.236	313.2366	2.251337882	1.170782593	0.00	1.464790588	up
2,6-Dihydroxypurine	4.651	153.04048	3.73403642	1.900735999	0.00	1.465690401	up
LPE 18:0	10.302	482.32367	0.346580334	-1.528738303	0.000109353	1.424058969	down
LPE 20:4	9.051	502.29211	3.236972848	1.694645264	0.000120958	1.425371201	up
P-Aminohippuric Acid	4.941	195.07617	0.100381808	-3.31643026	0.000131728	1.482190892	down
2,6-Di-tert-butyl-1,4-benzoquinone	7.173	221.15096	2.82934063	1.500465876	0.000146799	1.43230271	up
N4-Acetylcytidine	4.818	286.10284	0.280216222	-1.835387619	0.000152572	1.450881323	down

2,3-dihydroxypropyl 12-methyltridecanoate	8.107	285.24188	3.036584493	1.602449513	0.000158771	1.528143219	up
11(Z),14(Z),17(Z)-Eicosatrienoic acid	10.457	307.26248	6.220829496	2.637106965	0.000161055	1.473799533	up
3-methyl-5-oxo-5-(4-toluidino)pentanoic acid	1.264	258.10971	0.260072019	-1.943016906	0.000165678	1.468839221	down
PC (14:1e/3:0)	9.277	508.33954	0.442581418	-1.175985215	0.00016845	1.4232406	down
4-Acetamidobutyric Acid	4.73	146.08092	0.209733602	-2.253370075	0.000172095	1.422585538	down
N-Acetyl-D-tryptophan	5.536	247.10742	0.18145564	-2.462311193	0.00017597	1.431323606	down
(5-L-Glutamyl)-L-Amino Acid	1.35	219.09711	1.906220125	0.930714727	0.000190273	1.408834426	up
Isoquinoline	5.355	130.06496	0.144047807	-2.795380401	0.000191294	1.442691086	down
Coenzyme Q2	10.156	319.19397	2.053386742	1.038005375	0.00019373	1.463507186	up
1-Methyladenosine	1.354	282.11938	0.321803007	-1.635750286	0.000207608	1.402196396	down
N-[2-chloro-6-(trifluoromethoxy)phenyl]-2,2-dimethylpropanamide	1.25	296.06561	0.335257866	-1.576656913	0.000221832	1.450756279	down
Rosmarinic acid	4.96	356.13336	0.156034314	-2.680064762	0.000228669	1.44149653	down
Adenosine 5'-	1.347	348.0697	0.17012	-	0.00023	1.40906	down

monophosphate			4792	2.555334699	1793	7272	
4-oxo-5-phenylpentanoic acid	5.501	175.07515	0.219872637	-2.185260021	0.000234493	1.450527685	down
SDMA	1.3	203.15005	0.348915995	-1.519048359	0.00023488	1.409112735	down
6ß-Hydroxytestosterone	7.699	305.20822	2.074777029	1.052956302	0.00023502	1.498865629	up
Xanthosine	4.661	285.08252	4.481858115	2.164096977	0.000239396	1.452537944	up
6-Methoxy-2-naphthoic acid	5.506	203.07013	0.233136942	-2.10075047	0.000242244	1.425151241	down
ACar 16:1	6.88	416.33655	2.137007535	1.095591995	0.000253027	1.402367939	up
S-Adenosyl-L-methionine	1.173	399.14401	0.401276713	-1.31733066	0.000275711	1.401371757	down
1,3-dipyridin-3-ylpropane-1,3-dione	5.394	227.08118	0.071914439	-3.79757473	0.000285654	1.458762346	down
Ethyl chrysanthemumate	8.34	197.15344	2.50903013	1.327129796	0.000308955	1.454374193	up
Docosapentaenoic acid	10.267	331.26239	6.991621625	2.805627111	0.000309337	1.433186197	up
Creatinine	1.255	114.066	0.270296077	-1.887387521	0.000323031	1.42142602	down
5-Hydroxyindole-3-acetic acid	5.145	192.06532	0.327591538	-1.610030005	0.000351757	1.380291159	down
methyl 3,4,5-trihydroxycyclohex-1-ene-1-carboxylate	5.176	211.05742	0.376387196	-1.409710543	0.000361325	1.401529879	down
11-Deoxy prostaglandin F1β	10.175	323.25674	2.438958986	1.286265498	0.000367285	1.372582572	up
tetranor-12(R)-HETE	8.422	227.2003	2.501710067	1.3229146	0.000372722	1.394442976	up

16-Heptadecyne-1,2,4-triol	7.918	307.22375	2.902799588	1.537444971	0.000395014	1.386894097	up
8,8-dimethyl-2-phenyl-4H,8H-pyrano[2,3-h]chromen-4-one	4.732	305.12018	0.278703839	-1.843195222	0.000419313	1.382511139	down
Glycerophospho-N-palmitoyl ethanolamine	9.455	454.29196	0.369776494	-1.435274578	0.000529211	1.448478204	down
Glu-Glu	1.35	277.10245	2.085006564	1.060051925	0.000534289	1.387026557	up
LPE 18:1	9.473	480.30762	1.896072385	0.923014042	0.000558377	1.370553786	up
1-Methylhistidine	1.17	170.09218	0.392314025	-1.349919182	0.000569704	1.364531403	down
(2,6-dimethylmorpholino)(1-methyl-5-nitro-1H-pyrazol-4-yl)methanone	5.504	307.07822	0.115300296	-3.116531872	0.000580185	1.444592148	down
Spermidine	0.994	146.16498	0.191115798	-2.387481052	0.000590869	1.37209565	down
Heptadecanoic Acid	8.555	293.24701	2.325010072	1.217236966	0.000600554	1.449988204	up
Methyl 3-indolyacetate	5.81	190.08592	2.285990308	1.192819287	0.0006413	1.406959114	up
NAD+	1.858	332.56104	0.281352959	-1.829546961	0.000705887	1.352760003	down
(R)-Equol	5.493	243.1012	0.130527852	-2.937570415	0.000785456	1.416280387	down
Nicotinuric acid	5.165	181.0607	0.118415975	-3.078064372	0.000807783	1.431028376	down
3'-Adenosine monophosphate (3'-AMP)	5.078	348.06979	0.15330466	-2.705526548	0.000814101	1.417547947	down

Betaine	1.297	118.08606	2.706115116	1.436223212	0.00084384	1.354113807	up
Delta-Tridecalactone	8.935	213.18456	2.056948326	1.040505551	0.000962104	1.374799095	up
Hydrocortisone	5.995	363.21588	2.132683304	1.092669747	0.001029812	1.424190379	up
10-Undecenoic acid	8.304	185.15331	2.050580252	1.036032206	0.001046875	1.384738775	up
Palmitoyl ethanolamide	6.093	300.28912	3.607094239	1.850837116	0.001126021	1.339181346	up
N-Isovalerylglycine	5.227	160.09659	0.145127107	-2.784611087	0.001171791	1.404967359	down
Linoleoyl ethanolamide	9.313	324.28903	0.438090374	-1.190699578	0.001179219	1.327816422	down
5α-Pregnan-3,20-dione	9.897	317.24677	3.239274829	1.695670876	0.001216113	1.364039999	up
LPC 18:3	8.414	518.32379	0.478658894	-1.062930177	0.001383075	1.304685028	down
Testosterone	6.846	289.21576	0.357500049	-1.483984656	0.001394173	1.410299281	down
4-methoxy-6-(prop-2-en-1-yl)-2H-1,3-benzodioxole	5.42	193.08321	0.129107119	-2.953359541	0.001469307	1.379691805	down
5-Hydroxytryptophan	4.896	221.09181	0.381126903	-1.391656646	0.00147523	1.352059382	down
Cyclic ADP-ribose	1.356	542.06799	0.318052599	-1.652662721	0.001671956	1.30153735	down
2-Arachidonoyl glycerol	8.865	361.27295	2.617446677	1.388160147	0.001850433	1.338583348	up
N-Acetyl-L-tyrosine	5.346	224.09134	0.34985336	-1.515177746	0.002261031	1.309691049	down
N-Formylkynurenine	4.814	237.08665	0.110590282	-3.176703483	0.002264652	1.375486252	down

gamma-Glutamylglutamine	1.344	276.11847	1.991605298	0.993931758	0.00242049	1.268303787	up
L-Kynurenine	4.709	209.09178	0.348542319	-1.520594261	0.002493312	1.362771712	down
Cytidine	1.349	244.09258	1.993291836	0.995152949	0.002705111	1.364613508	up
2,4-dichlorobenzaldehyde 1-methyl-1-(6-methylpyridazin-3-yl)hydrazone	1.338	317.03406	2.444101944	1.289304462	0.003149345	1.344967169	up
N6,N6,N6-Trimethyl-L-lysine	1.142	189.15955	0.618254599	-0.693727028	0.003297048	1.250317945	down
Dl-Lanthionine	1.341	209.05969	2.107200016	1.075327261	0.003397946	1.324792319	up
L-Glutamic acid	1.268	148.06015	0.538386443	-0.893286014	0.003518496	1.308249105	down
Thr-Leu	1.116	233.14926	2.636739121	1.398754838	0.003885392	1.237453427	up
Methionine	1.966	150.05806	0.431522823	-1.212491229	0.003964888	1.298630914	down
Adenylosuccinic acid	4.733	464.08072	0.269900201	-1.889502044	0.004355225	1.221215901	down
Homo-Gamma-Linolenic Acid (C20:3)	10.176	307.26248	1.507342515	0.592007279	0.004523807	1.209369999	up
Cytidine 5'-monophosphate (hydrate)	1.352	324.05856	0.537153799	-0.896592873	0.004533934	1.238036864	down
APK	10.778	337.18121	1.615570437	0.692043652	0.004588102	1.216982009	up
Eicosapentaenoic acid	8.246	309.24124	1.704937251	0.769718643	0.004744734	1.280957177	up
ACar 15:0	6.933	386.32559	2.558705009	1.35541383	0.004926124	1.222572944	up

3-(4-nitrophenyl)[1,2,3]triazolo[1,5-a]quinazolin-5-amine	5.719	307.09372	2.191292207	1.131781879	0.005015178	1.246928257	up
N6-Acetyl-L-lysine	1.349	172.09656	0.48190274	-1.05318609	0.005580634	1.27457215	down
3,4-Dihydroxyphenylpropionic acid	5.137	205.047	0.533678354	-0.905957599	0.005683823	1.198330654	down
o-Cresol	5.313	109.06466	0.310775352	-1.686056007	0.005965354	1.272577219	down
Cinnamoylglycine	5.629	206.08101	0.250318991	-1.998160347	0.006022355	1.280408424	down
L-Aspartic acid	1.248	134.04463	0.439538878	-1.185937314	0.006025898	1.234440941	down
Monoolein	10.182	357.29953	2.373314184	1.2469031	0.006083893	1.187485435	up
Methyldopa	5.647	212.09143	0.478129492	-1.064526699	0.006411575	1.186161534	down
N-Methylhydantoin	1.371	115.05001	1.623742373	0.699322749	0.006684775	1.207004614	up
(2R,3S,4S,5R,6S)-2-(hydroxymethyl)-6-phenoxyoxane-3,4,5-triol	5.295	296.10992	0.282788071	-1.822206832	0.007008854	1.257198315	down
Michler's ketone	6.789	269.16446	0.254680698	-1.97323847	0.007061911	1.273301809	down
Xanthine	2.161	153.04051	1.980370189	0.985770137	0.007415661	1.28714023	up
Nicotinamide adenine	1.353	664.11566	0.311629791	-1.6820	0.00949663	1.156338643	down

dinucleotide (NAD+)				94936			
Glycyl-L-leucine	1.352	189.12314	0.364916086	-1.454363347	0.010058799	1.206616924	down
5-Methyl-dl-tryptophan	5.415	219.11259	0.478075081	-1.064690887	0.011535515	1.123478389	down
Phosphocholine	1.253	184.07324	0.582550584	-0.779544768	0.011834474	1.135999722	down
4-[4-(trifluoromethoxy)anilino]-2H-1,3-benzoxazin-2-one	4.67	323.069	0.427903958	-1.22464107	0.012982193	1.14281363	down
DL-m-Tyrosine	5.5	164.07034	0.392158619	-1.350490785	0.01317084	1.186751212	down
(2R)-2-[(2R,5S)-5-[(2S)-2-hydroxybutyl]oxolan-2-yl]propanoic acid	5.751	217.14311	0.651004317	-0.619260984	0.013540262	1.111441643	down
Leucylproline	5.018	229.15442	2.054014647	1.038446469	0.014644875	1.188099338	up
4-morpholinobenzoic acid	5.552	208.09676	0.420064694	-1.25131656	0.01659673	1.140621888	down
bicyclo[2.2.2]oct-2-en-1-yl 4-methylbenzene-1-sulfonate	4.804	279.1004	0.380248306	-1.394986273	0.017265647	1.102732647	down
MAG (18:2)	9.619	355.28345	1.690703369	0.757623564	0.021743773	1.126920218	up
4-oxododecanedioic acid	3.924	262.16449	0.585407009	-0.772488075	0.02377316	1.038351634	down
Dl-Indole-3-lactic acid	5.543	206.08121	0.616057496	-0.698863091	0.024499544	1.056569927	down

Pilocarpine	7.893	171.17377	1.500970725	0.585895839	0.024738659	1.040069594	up
Dehydrocholic acid	10.529	403.24936	2.409851935	1.268944508	0.024902867	1.099895944	up
Decanoylcarnitine	5.899	316.24796	1.68044869	0.748846493	0.025823137	1.021062305	up
Cytidine 5'-diphosphocholine	1.3	489.11462	0.594079657	-0.751271708	0.02660095	1.028635109	down
PC (14:0e/2:0)	9.276	496.3389	0.492593144	-1.021531548	0.027264619	1.085369771	down
Thymine	1.304	127.05009	1.842849011	0.881937873	0.028040588	1.025290541	up
Stearamide	10.638	284.29404	0.434284403	-1.203287954	0.03027247	1.072775208	down
5-allyl-4,6-dimethyl-2-oxo-1,2-dihydropyridine-3-carbonitrile	5.792	189.10202	5.145555261	2.363326768	0.037076627	1.032055476	up

1) "Up-down regulation" refers to the up- or down-regulation of metabolites in Angus cattle PF compared with that in Simmental cattle PF.

Table S2. Differential metabolites of perirenal fat between Angus cattle and Simmental cattle(Negative ion mode).

**Name**	**RT [min]**	**m/z**	**FC**	**log2FC**	**Pvalue**	**VIP**	**Up.Down**
11-Ketoetiocholanolone	8.034	303.1965	11.36031	3.50593	0.00	1.429908	up
N-benzyl-N-isopropyl-N'-[4-(trifluoromethoxy)phenyl]urea	9.407	351.1269	13.01135	3.701699	0.00	1.435199	up
Bicyclo Prostaglandin E2	7.446	333.2074	11.40017	3.510984	0.00	1.435747	up

LPE 22:5	9.116	526.2937	3.466895	1.793644	0.00	1.410114	up
13,14-dihydro-15-keto-tetranor Prostaglandin D2	6.941	279.16	5.291901	2.403786	0.00	1.413042	up
Uridine 5'-diphosphogalactose	1.632	565.0474	0.125071	-2.99918	0.00	1.428931	down
FAHFA (20:3/22:5)	10.498	633.4868	22.02794	4.461263	0.00	1.403448	up
LPE 20:5	8.39	498.2623	9.392475	3.231505	0.00	1.398659	up
Gluconolactone	1.335	177.04	0.477821	-1.06546	0.00	1.42586	down
Uridine diphosphate glucose	1.492	565.0476	0.060218	-4.05367	0.00	1.404002	down
Lysops 22:5	9.266	570.2811	7.71538	2.947737	0.00	1.401617	up
Prostaglandin H2	8.256	333.2046	2.45555	1.296046	0.00	1.389015	up
15(R)-Lipoxin A4	7.934	351.2153	2.869557	1.520828	0.00	1.383294	up
3-Oxo-7alpha,12alpha-hydroxy-5beta-cholanoic acid	6.555	405.2644	4.554347	2.187244	0.00	1.397485	up
(±)12(13)-DiHOME	8.697	313.2385	2.074403	1.052696	0.00	1.383908	up
Hexanoylglycine	5.494	172.0976	0.207614	-2.26802	0.00	1.38622	down
Lysops 22:6	8.888	568.2653	4.069079	2.024702	0.00	1.363812	up
1-(4-methylphenyl)-3-(2-pyridylthio)pyrrolidine-2,5-dione	3.914	298.0696	7.502938	2.907456	0.00	1.395572	up
FAHFA (14:0/16:2)	9.659	477.3923	2.905206	1.538641	0.00	1.366606	up

L-Arabinitol	1.298	151.0609	0.483554	-1.04825	0.00	1.380839	down
Gluconic acid	1.299	195.0506	0.382003	-1.38835	0.00	1.39076	down
23-Norcholic acid	6.822	393.2645	1.7003	0.765789	0.00	1.345482	up
N2-(2-methoxyphenyl)-5-chloro-3-methylbenzo[b]thiophene-2-sulfonamide	5.52	366.0045	0.141131	-2.82489	0.00	1.332919	down
D-Ribose	1.299	195.0508	0.409151	-1.28929	0.00	1.387471	down
Cholic acid	7.316	407.28	4.304135	2.105723	0.00	1.389013	up
Nicotinamide adenine dinucleotide phosphate	1.437	742.0677	0.163813	-2.60988	0.00	1.31369	down
Androsterone	9.764	289.2173	7.021071	2.811691	0.00	1.398637	up
13-Hpotre(R)	7.528	309.2071	1.966967	0.975973	0.00	1.330911	up
2-(Formylamino) Benzoic Acid	5.671	164.0349	0.542706	-0.88176	0.00	1.338133	down
Lysopc 14:0	9.924	466.2934	0.29141	-1.77888	0.00	1.317946	down
gamma-Glutamylleucine	5.281	259.1297	0.321834	-1.63561	0.00	1.30831	down
Lauric acid ethyl ester	9.66	227.2024	4.149556	2.052957	0.00	1.334584	up
N-Tigloylglycine	5.187	156.0663	0.113542	-3.13871	0.00	1.368604	down
Jasmonic acid	6.094	209.118	0.248432	-2.00908	0.00	1.378916	down
Porphobilinogen	4.873	225.0879	0.443481	-1.17306	0.00	1.330799	down
Corticosterone	9.664	345.2046	5.031708	2.331048	0.00	1.377765	up
Levodopa	5.952	196.0612	1.846636	0.8849	0.00	1.370753	up

4-Aminobutyric acid	1.285	102.0558	0.405418	-1.30252	0.000102	1.331099	down
1,4-Cyclohexanedicarboxylic acid	5.49	171.0661	0.349867	-1.51512	0.000117	1.366922	down
Uridine 5’-diphosphate-D-glucuronate	1.837	579.0264	0.251498	-1.99138	0.000127	1.282372	down
Myristic acid	9.533	227.2023	4.139862	2.049583	0.000156	1.358679	up
Capric acid	7.693	171.1387	3.408944	1.769325	0.000157	1.324057	up
LysoPE 18:0	9.531	480.31	0.262878	-1.92753	0.000161	1.278659	down
Ergothioneine	1.347	228.0806	2.758118	1.463684	0.00018	1.320963	up
2,3-dinor Prostaglandin E1	7.483	307.1915	1.992517	0.994592	0.000225	1.349409	up
2-(acetylamino)-3-(1H-indol-3-yl)propanoic acid	5.556	245.0929	0.185256	-2.43241	0.000283	1.318222	down
Guanosine monophosphate (GMP)	1.439	362.0508	0.29229	-1.77453	0.000309	1.25706	down
Phenylacetylglycine	5.435	192.0664	0.100927	-3.30861	0.000312	1.346515	down
Epoxomicin	9.553	553.3619	0.155652	-2.6836	0.000336	1.294015	down
4-Methylphenol	5.75	107.05	0.16064	-2.6381	0.000401	1.327927	down
Galacturonic acid	5.352	193.035	0.124853	-3.00169	0.000424	1.32238	down
Uridine monophosphate (UMP)	1.758	323.0286	0.267768	-1.90094	0.000508	1.282084	down
Cytidine-5'-monophosphate	1.348	322.0445	0.547808	-0.86826	0.000532	1.224109	down
3-(2,4-dichlorophenyl	5.435	274.989	0.073531	-3.7655	0.000587	1.328459	down

)pentanedioic acid							
L-Adrenaline	5.541	182.082	0.1347	-2.89218	0.000611	1.309443	down
FAHFA (16:1/18:3)	9.889	529.4233	4.106105	2.037771	0.00064	1.300622	up
Inosine 5'-Monophosphate	1.474	347.0394	0.303287	-1.72124	0.000891	1.280733	down
LPC 20:4	8.897	588.3304	2.754154	1.461609	0.001099	1.248933	up
LPA 6:0	6.181	269.0796	0.294159	-1.76533	0.001313	1.214852	down
Allantoin	1.325	157.0363	0.28743	-1.79872	0.001338	1.278074	down
methyl {[(2-oxo-2H-pyran-6-yl)carbonyl]amino}methanethioate	5.322	212.0019	0.128877	-2.95593	0.001362	1.264887	down
Indole-3-lactic acid	5.557	204.0664	0.521248	-0.93996	0.001475	1.272749	down
UDP-N-acetyl-alpha-D-glucosamine	1.487	606.0744	0.425407	-1.23308	0.001563	1.190478	down
Homovanillic acid	5.152	181.0505	0.46305	-1.11076	0.001705	1.261912	down
N-Acetylvaline	5.237	158.0819	0.141539	-2.82073	0.001737	1.269656	down
8Z,11Z,14Z-Eicosatrienoic acid	10.503	305.2485	6.190976	2.630167	0.001769	1.257591	up
2-{1-[2-(4-benzhydrylpiperazino)-2-oxoethyl]cyclopentyl}acetic acid	10.492	419.2289	4.88191	2.287446	0.001799	1.269747	up
Adenosine diphosphate (ADP)	1.514	426.0219	0.580325	-0.78507	0.001849	1.194166	down
2-Methylbutyl beta-D-	4.987	295.1397	0.30583	-1.7092	0.00203	1.252721	down

glucopyranoside							
trans-10-Heptadecenoic Acid	10.342	267.2328	4.325479	2.11286	0.002083	1.260318	up
Pregnenolone	9.953	315.2327	3.396514	1.764055	0.002436	1.246185	up
LPC 20:3	9.499	590.3461	3.793346	1.923471	0.002996	1.199843	up
Docosahexaenoic Acid	9.995	327.2329	4.126143	2.044794	0.005004	1.195344	up
4-oxo-4-(4-toluidino)but-2-enoic acid	5.645	204.0663	0.225839	-2.14663	0.005461	1.179485	down
(±)11(12)-EET	8.264	319.2273	1.942574	0.95797	0.005601	1.098547	up
Uridine	2.321	243.0621	1.554276	0.636242	0.00577	1.105016	up
LPC 14:0	8.593	512.2992	0.562211	-0.83082	0.005868	1.081405	down
Hippuric acid	5.326	178.0506	0.270126	-1.8883	0.00611	1.179954	down
Adenosine diphosphate ribose	1.438	558.0642	0.239321	-2.06298	0.006316	1.066254	down
N-Glycolylneuraminic acid	1.326	278.0876	0.486676	-1.03897	0.006666	1.116082	down
LPE 16:0	9.487	452.2784	0.436808	-1.19493	0.006873	1.181413	down
Nicotinamide adenine dinucleotide	1.357	662.1018	0.328908	-1.60425	0.007465	1.058496	down
LPE 17:0	9.799	466.2934	0.388284	-1.36482	0.008698	1.11657	down
Arachidonic acid	10.085	303.2327	2.978932	1.574795	0.009398	1.146129	up
Elaidic acid	10.793	281.2484	3.572857	1.837078	0.010258	1.128838	up
all-cis-4,7,10,13,16-Docosapentaenoic acid	10.489	329.2486	3.589267	1.843689	0.010364	1.10055	up
6-Keto-	6.296	369.2281	1.7059	0.77054	0.01210	1.03275	up

prostaglandin f1alpha			09	1	3	4	
FAHFA (18:2/20:4)	10.192	581.4547	3.176275	1.667336	0.012217	1.116564	up
(±)9(10)-EpOME	7.906	295.2275	1.658051	0.729489	0.01332	1.06459	up
Deoxycholic Acid	8.185	391.2852	2.390759	1.257469	0.013633	1.01715	up
L-Ascorbic acid 2-sulfate	1.489	254.9813	0.391435	-1.35316	0.014323	1.02667	down
7-Hydroxy-3,4-dihydrocarbostyril	5.498	162.0557	0.392364	-1.34973	0.014718	1.057362	down
N-Acetyl-α-D-glucosamine 1-phosphate	1.412	300.0486	1.671869	0.741462	0.017124	1.014385	up
Glu-Gln	1.343	274.1042	1.849424	0.887076	0.01749	1.008458	up
Hydroquinone	5.088	109.0293	0.473967	-1.07714	0.018031	1.046403	down
Asp-glu	1.366	261.0729	1.50416	0.588958	0.01941	1.039474	up
Tetrahydrocorticosterone	10.085	349.2381	3.635635	1.862207	0.019757	1.067507	up
Palmitic acid	10.574	255.2329	2.456272	1.29647	0.020628	1.048935	up
FAHFA (16:0/18:2)	10.576	533.4548	2.022083	1.015842	0.031126	1.008076	up
